# Successful treatment of a colonic cyst by endoscopic aspiration and sclerotherapy

**DOI:** 10.1055/a-2068-8372

**Published:** 2023-04-26

**Authors:** Zhang Tao, Jie Liu, Dan Hu, Ya Lan Chen, Feng Li, Feng Ying Lin, Shenggang Feng

**Affiliations:** Department of Gastroenterology, Nanchong Central Hospital, The Second Clinical Medical College, North Sichuan Medical College, Nanchong City, Sichuan, China

A 38-year-old man presented to our department with constipation that had persisted for 2 years. Upon colonoscopy, we discovered a smooth protrusion (about 2 cm × 2 cm) at the hepatic flexure of his colon ( Fig. 1 ). Anechoic ultrasonic colonoscopy was suggestive of a cyst. A transparent puncture needle was used to puncture into the cyst cavity and a syringe was used to aspirate the cyst fluid ( Video 1 ). After sufficient aspiration, we verified the disappearance of the cyst ( Fig. 2 ) and 2 ml of yellow cyst fluid ( Fig. 3 ). Subsequently, 2 ml of polidocanol-methylene blue mixture was injected into the cyst cavity through the puncture needle ( Video 1 ). A 6-month follow-up colonoscopy revealed no cyst at the hepatic flexure of the patient’s colon ( Fig. 4 , Video 1 ).

**Fig. 1:**
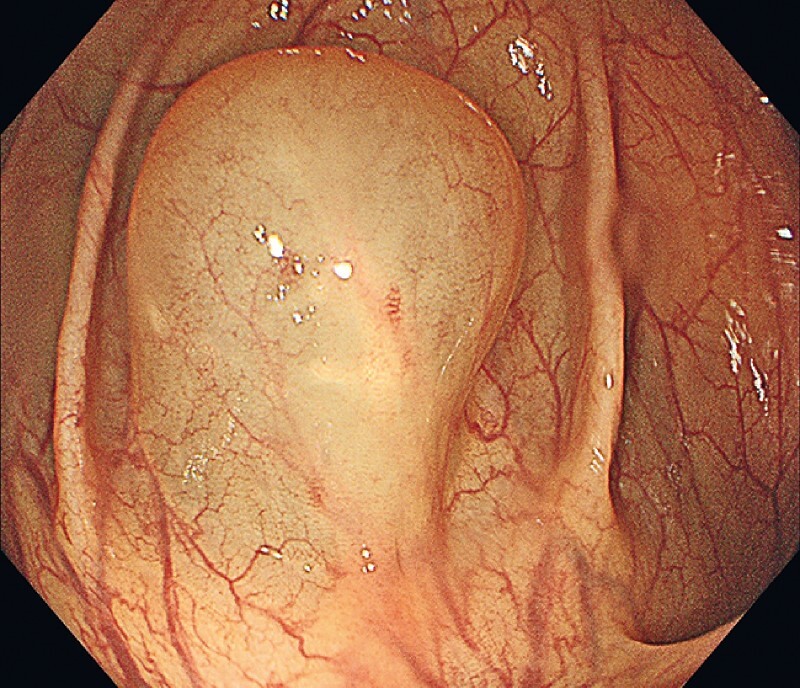
A smooth protrusion at the hepatic flexure of the patient’s colon.

**Video 1** Successful treatment of a colonic cyst by endoscopic aspiration and sclerotherapy.


**Fig. 2:**
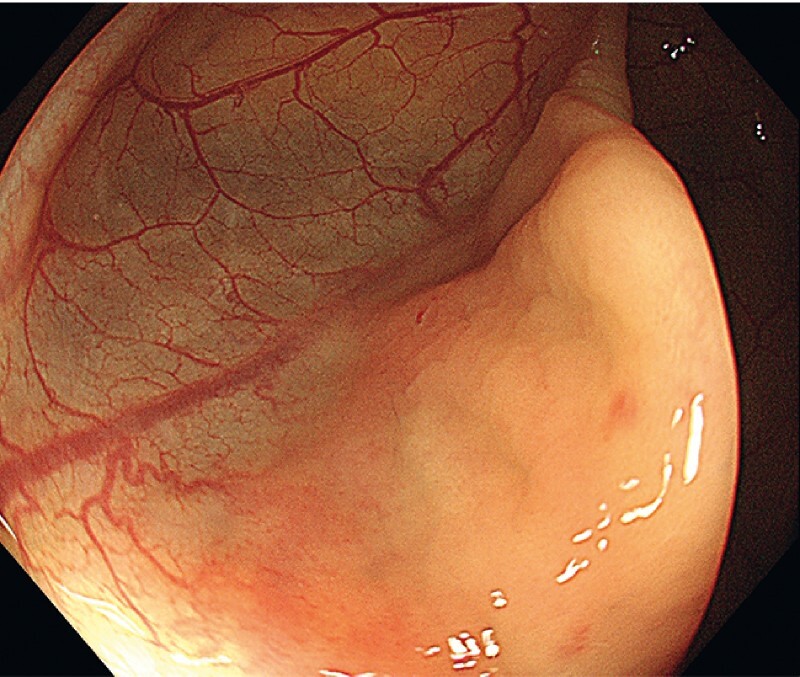
Disappearance of the cyst after endoscopic aspiration.

**Fig. 3:**
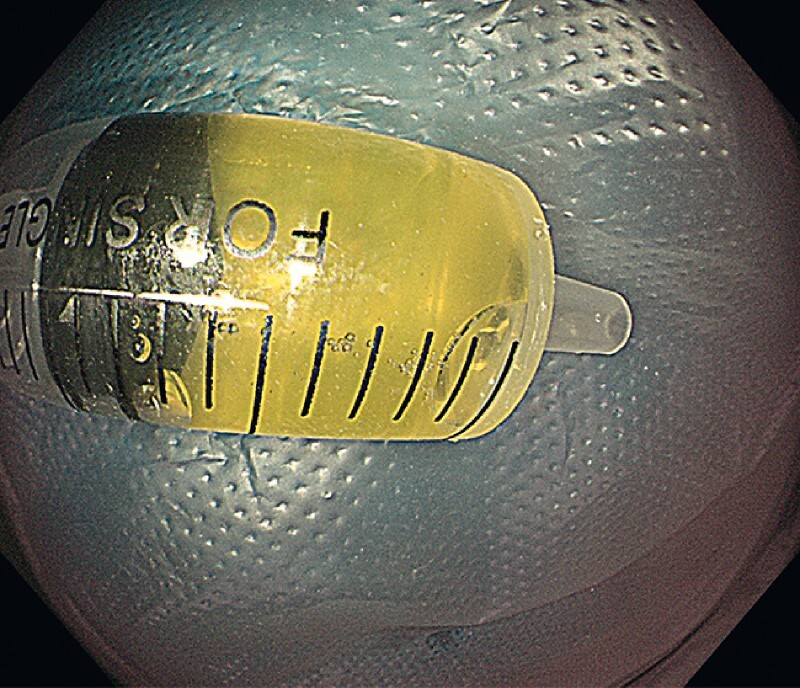
Yellow cyst fluid.

**Fig. 4:**
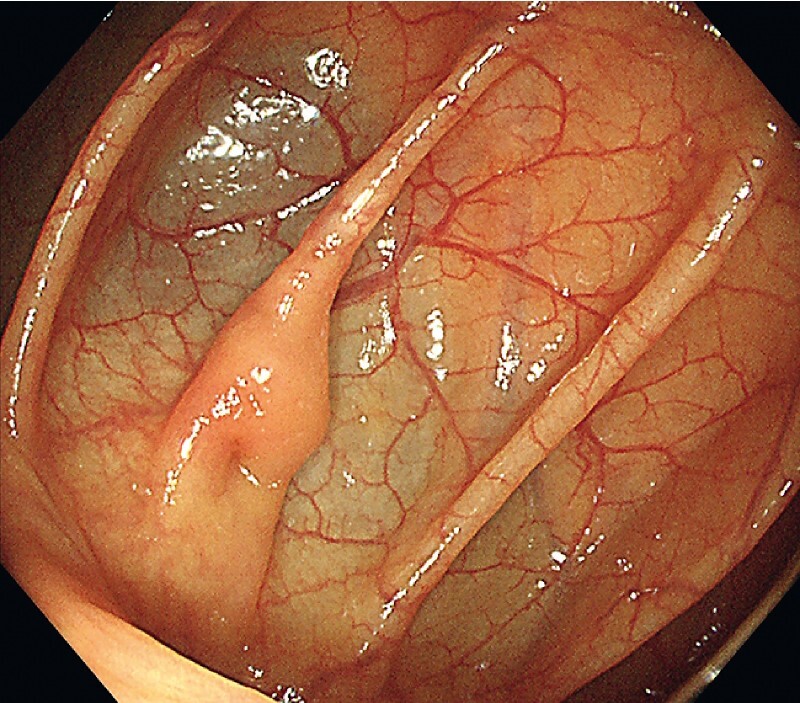
No cyst at the hepatic flexure of the colon after 6 months.

Colonic cysts are benign lesions associated with chronic constipation and abdominal pain 1 . However, colonic cysts must be surgically removed if they become large to prevent intestinal obstruction or intussusception 1 2 . Aspiration plus sclerotherapy is considered a safe and effective treatment for simple hepatic cysts 3 . We are unsure whether such therapy would be safe and effective for a colonic cyst because of the thinness of colon tissue and possibility of perforation. Herein, we reported the successful and uneventful aspiration and sclerotherapy of a colonic cyst.

Endoscopy_UCTN_Code_CCL_1AD_2AI

## Notes

Endoscopy E-Videos

https://eref.thieme.de/e-videos
*E-Videos* is an open access online section of the journal *Endoscopy* , reporting on interesting cases and new techniques in gastroenterological endoscopy. All papers include a high-quality video and are published with a Creative Commons CC-BY license. Endoscopy E-Videos qualify for HINARI discounts and waivers and eligibility is automatically checked during the submission process. We grant 100% waivers to articles whose corresponding authors are based in Group A countries and 50% waivers to those who are based in Group B countries as classified by Research4Life (see: https://www.research4life.org/access/eligibility/ ). This section has its own submission website at https://mc.manuscriptcentral.com/e-videos
